# What factors condition the financial viability of sheltered employment centres? Empirical evidence

**DOI:** 10.1007/s11846-021-00450-3

**Published:** 2021-03-03

**Authors:** Vera Gelashvili, María-Jesús Segovia-Vargas, María-del-Mar Camacho-Miñano

**Affiliations:** 1 Department of Business Economics, Faculty of Legal and Social Sciences, King Juan Carlos University, Paseo de los Artilleros s/n, 28032 Madrid, Spain; 2 Department of Financial and Actuarial Economics & Statistics, Faculty of Economics and Business, Complutense University of Madrid, Campus de Somosaguas, Pozuelo de Alarcón, 28223 Madrid, Spain; 3 Accounting & Finance Department, Faculty of Economics and Business, Complutense University of Madrid, Campus de Somosaguas, Pozuelo de Alarcón, 28223 Madrid, Spain

**Keywords:** Sheltered employment centres, Profitability, Economic viability, Panel data, Decision trees, J58, M4, M41

## Abstract

Nowadays, employment is a challenge for people but more for disabled ones. Prior literature shows that, at a European level, there are different ways for people with disabilities to find a job, such as a quota system, sheltered workshops, supported employment, etc. In Spain, sheltered employment centres are prototypes of sheltered workshops aimed at integrating more people with disabilities into the workplace. This research project aims to give visibility to these firms and to gain an understanding of their economic and financial situation. Using the whole sample of sheltered employment centres in Spain, and their financial data from 2004 to 2016, we show which variables explain their viability. Additionally, in light of the imminent worldwide crisis due to the COVID-19 pandemic situation, we want to test the impact of the last economic crisis on the profitability of sheltered employment centres. The main contribution of this study is that the size of these companies, age, financial risk and sales growth, are determining factors for their profitability. And, the economic and financial crisis has conditioned the viability of sheltered employment centres as many firms on the market registered a decrease in their profitability in the years following the crisis but survived. This study helps to shed light on the economic and financial situation of this kind of firms as well as their social visibility.

## Introduction

Employment plays a central role in the lives of people with disabilities, offering not only monetary rewards but also such benefits as social identity, contacts and support (Shepherd 1989). Some researchers believe that creating opportunities for members of this population to get and keep jobs has a more profound effect on more areas of their lives than any other medical or social intervention (Boardman et al. 2003). Around Europe, there are a variety of programs that support and promote the employment of people with disabilities. One of the ways of combating labour discrimination against people with disabilities is through social firms (Cooney et al. 2016). Social enterprises put social objectives above economic ones, and if the enterprise is profitable, profits are often reinvested to promote the company's major social objectives (Del Negro 2012). However, these companies are not well known, which means that the great work they do is not always recognized. Within the category of social enterprises, are included sheltered employment centres (Díaz-Foncea and Marcuello 2012), companies that promote labour and social integration of people with disabilities in Spain.

Sheltered employment centres are special firms because their workforce is made up of at least 70% of people with disabilities, according to the Spanish law for disabled people (Royal Decree 2273/1985, approving the regulations on sheltered employment centres as defined in article 42 of Law 13/1982, of 7 April 1982, on social integration of people with disabilities). They are important companies for society and, especially, for disabled people due to the elimination of labour and social inclusion barriers (Calvo 2004; Mendoza et al. 2019). The role played by sheltered employment centres in the social economy of our country is important because almost 7% of social firms are made up of those companies (CEPES 2020). Furthermore, sheltered employment centres have more stable jobs for workers with disabilities than ordinary companies (Rodríguez and Cueto 2013).

The role, evolution, importance, professionalisation, wage differentials and other global aspects of sheltered employment centres have been studied for many years (Visier 1998; Rubio 2003; Laloma 2007; Jordán de Urríes and Verdugo 2010; Rodríguez et al. 2012; López et al. 2014; Manzano Martín et al. 2016; Gelashvili et al. 2015a,b; Monzón-Campos and Herrero-Montagud 2016; Gelashvili et al. 2018; Mendoza et al. 2019) but their economic and financial aspects have not been investigated in depth. In the last five years, some empirical studies have analysed the level of economic impact generated by sheltered employment centres in different regions of Spain (López et al. 2014; Manzano Martín et al. 2016; Gelashvili et al. 2015a) but none of them used data for all sheltered employment centres of Spain.

Apart from the higher number of workers with disabilities in sheltered employment centres, there is another characteristic differentiating them from normal companies. Sheltered employment centres can receive public subsidies. According to Law 13/1982, of 7 April (LISMI), sheltered employment centres receive public subsidies for the labour insertion of people with disabilities. Those subsidies provided to these entities represent important economic flows for them (López et al. 2014). The purpose of the received subsidies is different: discounts for companies’ social security contributions, subsidies to adapt workstations, new investments, creation of new workplaces, etc. (Mallender et al. 2015). Thus, some authors point out that these public subsidies could determine their success in the labour market (Laloma 2007; Jordán de Urríes and Verdugo 2010). However, there are no empirical studies that test this affirmation.

Other studies on sheltered employment centres have examined the evolution of these companies and have concluded that their numbers continued to grow even during the economic crisis that hit Spain in 2008 and beyond (Camacho-Miñano and Perez 2012; Gelashvili et al. 2015b). Meanwhile, in Spain, the number of SMEs declined. According to comparative data of SMEs, from 2006 to 2015 the percentage decrease was − 3.02, almost 101,000 SMEs less than in 2006.^1^

Bearing all of this in mind, this study aims to analyse which main factors determine the profitability of sheltered employment centres and test the main empirical assumptions about these specific firms such as the role of public subsidies or the impact of the economic crisis on their viability. The main contribution of this study is that the size, age, financial risk and sales growth are determining variables for the profitability of Spanish sheltered employment centres. Moreover, the economic crisis has negatively conditioned their viability. That means that the profitability of sheltered employment centres has decreased in the years following the crisis. Although their profitability has decreased in times of crisis, these companies have managed to stay in the market, since some studies have shown that the number of these companies has increased during and after the economic and financial crisis. This fact is an important contribution for these special companies, since the current situation caused by the COVID-19 has begun to destroy employment around the world and will grow even more among the most disadvantaged, who have the least likely to remain in the ordinary labour market, the people with disabilities. Therefore, the importance of these companies is going to increase in the period of the current crisis for people with disabilities.

This paper is organized as follows: the second section includes the literature review about sheltered employment centres in academic studies. The third section shows proposed research questions. The sample of the research, methodology and variables of the study are shown in the fourth section. The results of the financial data analysis and our main conclusions are presented in the fifth and sixth sections.

## Literature background

### Sheltered workshop and sheltered employment centres in Spain: Literature review

Over the years, much has been written about sheltered workshops (Whitehead 1979; Rosen et al. 1993; Visier 1998; Krupa et al. 2003; Migliore et al. 2007; Migliore 2010; Evert et al. 2012; Hoffman 2013; Dlouhy and Mitchell 2015; Mallender et al. 2015; Yell et al. 2017; Lukas et al. 2018). In the beginning, they were developed by charities or religious organizations (Migliore 2010), but then the tasks and definition of sheltered workshops changed (Malo 2003; Galer 2014). Sheltered workshops are defined as “entities which specifically employ disabled people and receive subsidies in compensation for the reduced productivity of their workforce” (Mallender et al. 2015). Sheltered workshops aim to help unemployed people with disabilities to “prepare” and become competitively employed within the community (Evert et al. 2012). With social and labour integration, the rehabilitation of people with disabilities is also the main issue for sheltered workshops (Visier 1998; Mallender et al. 2015).

Each country has its systems of employability and social/labour inclusion of people with disabilities (Visier 1998). For example, most European countries have quota obligation systems (Greve 2009), sheltered workshops, supported employment for people with disabilities (Egido et al. 2009; Mallender et al. 2015; Hoffmann and Richter 2019) which includes start-up support for entrepreneurship by people with disabilities (Renko et al. 2016), etc. In Spain, sheltered employment centres are prototypes of sheltered workshops aimed at integrating more people with disabilities into the workplace (Royal Decree 2273/1985, approving the regulations on sheltered employment centres as defined in Article 42 of Law 13/1982, of 7 April 1982, on the social integration of people with disabilities). Due to their economic and social importance, sheltered employment centres are referred to as social enterprises at the European level (López et al. 2014). Studies about sheltered employment centres in Spain analyse how the management of these enterprises works (Giménez 2012; López et al. 2014), what the main objectives of these firms are (Martínez 2009; Jordán de Urríes and Verdugo 2010) or how the number of employees with disabilities has grown during last years (Giménez 2012; Díaz-Foncea and Marcuello 2014; Penabad et al. 2019).

Although there are many studies on different aspects of sheltered employment centres (Calderón and Calderón 2012; Rodríguez et al. 2012; López et al. 2014; Penabad et al. 2019; Rodríguez 2017), there is a notable lack of literature on the economic and financial viability and profitability of these centres (Manzano Martín et al. 2016; Gelashvili et al. 2015a, b). Thus, it is important to know how the management of these centres works and what factors determine their economic and financial viability.

The first study on the profitability of sheltered employment centres was a study carried out by López et al. (2014). This study examines whether the level of economic impact generated by the sheltered employment centres depends on the kind of business activities carried out. The sample for the research included 66 sheltered employment centres of Aragón, another specific region of Spain. Their results have shown that the level of the economic impact of sheltered employment centres depends only on their business activities. Their social activities are not linked to their economic impact although the former are essential due to their objective.

The study carried out by Gelashvili et al. (2015a) examined 100 sheltered employment centres in the region of Madrid. The main objective of this paper was to know whether public subsidies were one of the main factors to determine their profitability. Their results showed that sheltered employment centres could be productive enterprises, on average, even without public subsidies.

Manzano Martín et al. (2016) studied 103 sheltered employment centres of Castilla-León, a region of Spain. Their principal findings show that in Castilla-León there are a large number of private sheltered employment centres, dominated especially by small and medium-sized centres. Their results showed that sheltered employment centres are able to obtain as many positive results as other ordinary companies.

To the best of our knowledge, there is no empirical study that examines the economic and financial viability of sheltered employment centres using all sheltered employment centres in Spain, which is the whole population in our country. At the same time, public subsidies are an important variable and differentiate them from an SME. For this reason, it is interesting to show their influence on its profitability and viability. Another issue is that there are additional subsidies in each of the autonomous communities in which each sheltered employment centre is located (Laloma 2007), so the results of previous research may change by using the financial data of all sheltered employment centres.

### Viability of firms

The viability of a company is studied based on its quantitative and qualitative data and the success or failure of a company depends on many factors. The profitability of enterprises is essential for their viability. Indeed, this topic about the factors that have an impact on the profitability of a company has been studied in some papers such as Schmalensee (1985), Rumelt (1991), Fernández et al. (1996); Claver et al. (2002) and González et al. (2002). According to Claver et al. (2002), the profitability of a firm depends on the resources and capabilities of each company, making them different from others, but not only because the management of these resources and capabilities is essential for determining their success or failure. Some other studies have pointed out that the profitability of companies depends on their employees’ capacity for teamwork, training, and skills (Claver et al. 2002; Rubio and Aragón 2002; Isaac et al. 2009), on their size (Suarez 2000) or the sector of their activities (Iglesias et al. 2007). Previous literature presents different economic and financial variables such as solvency, liquidation, borrowed funds, the share of external funding, asset turnover, sales margin, asset rotation, debt, funds generated by sales, etc. to evaluate the profitability of different types of companies (Fernández and García 1991; Fernández et al. 1996; Suarez 2000; González et al. 2002; Isaac et al. 2009). Fariñas and Rodríguez Romero (1986) analyse the future viability of companies from different countries and point out that their profitability depends on their nationality as well. Another variable that could also influence the profitability of the company is its level of corporate social responsibility (CRS) (Waddock and Graves 1997; Tsoutsoura 2004; Mahbuba and Farzana 2013). A recent study by Shahzad and Sharfman (2015) has detected a positive relationship between corporate social responsibility and the financial performance of firms.

There are some studies on the economic and financial viability and profitability of SMEs or even family enterprises (Claver et al. 2002; González et al. 2002; Luengo et al. 2005; Isaac et al. 2009). One of the main conclusions of those studies is that, for SMEs, profitability has become the most common financial indicator to measure the level of success or failure in business management (González et al. 2002). Moreover, the past economic and financial crisis impended the regular operation or even the survival of many companies (Isaac et al. 2009; Cowling et al. 2012; Carmona et al. 2013) and profitability is one of the most important variables for long-term survival in order to evaluate the yields of any company.

The studies analyzed above have shown the importance of social enterprises for the most vulnerable people in the market, in particular, the great work done by sheltered employment centres for people with disabilities even during the past economic and financial crisis. Recent studies indicate that we are at the beginning of a new economic and financial crisis almost all over the world because of the COVID-19 (Baldwin and Mauro 2020; Fernandes 2020). Several studies from different countries have estimated that the COVID -19 will lead to a catastrophic decrease in employment (Beirne et al. 2020; Blustein et al. 2020; Coibion et al. 2020). The latest data from the Public Service of State Employment in Spain has shown that unemployment is increasing month by month, the total number of job seekers in Spain currently stands at almost 4 million and the unemployment figures for April represent an increase of 7.97% compared to March.^2^ Due to the innumerable barriers for people with disabilities to access or keep employment in the ordinary companies (Gannon and Nolan 2004; Parker Harris et al. 2012), they are more likely to be unemployed and, if employed, to be paid less (Lang et al. 2011). For this reason, the study of sheltered employment centres in the times of the previous economic and financial crisis can help to shed light on how they can survive this global crisis that is coming.

## Research questions

Previous literature has been published about the influence of size, sector and location on profitability (Fariñas and Rodríguez Romero 1986; González Perez 1997 Suarez 2000; Sanchez and García 2003; Iglesias et al. 2006). Sanchez and García (2003) point out that competitive advantages help larger companies to obtain greater profitability, while the flexibility and adaptability of SMEs allow them to obtain a better performance. A study on integration companies shows that the success of these companies depends on their location and the sector of their activities (Retolaza et al. 2007). In addition, some studies have highlighted that public subsidies may play an important role in the economic viability of sheltered employment centres (Laloma 2007; Jordán de Urríes and Verdugo 2010). In the case of sheltered employment centres, it would be important to know the effect of these three important variables on their profitability. Thus, our research questions are presented as follows:

**RQ 1**: *What are the main financial factors that determine the profitability of sheltered employment centres?*

The past economic and financial crisis and its expansion over time force companies to survive in the current social and economic circumstances (Carmona et al. 2013). Despite the environmental crisis they are witnessing, sheltered employment centres are managing to create and maintain workplaces for people with disabilities (Manzano Martín et al. 2016). Also, the evolution of sheltered employment centres shows that the number of centres increased during the time of the crisis (Gelashvili et al. 2015b), although it punished many SMEs in Europe (Kokocinska and Rekowski 2013). From here, the next research question can be formulated:

***RQ 2***: *Has the economic and financial crisis conditioned the profitability of sheltered employment centres?*

It is important to analyze the impact of the previous crisis on these special companies since another one is coming and it is necessary to see how they are going to handle the situation so that people with disabilities be less affected.

The next section gives detailed information about the sample, variables and methodology of the study.

## Sample, variables and methodology

### Sample and data collection

According to data available for 2016, 1,834 sheltered employment centres represented all those existing in Spain. Through the Monitoring and Management Service for Supporting Employment of People with Disabilities assigned to the Employment Secretary (SEPE), it was possible to access the names of all existing sheltered employment centres at the end of 2016. There is no accessible database of all the sheltered employment centres in Spain on the internet because the employment competencies in our country are distributed in each region.

The SABI^3^ database was used for extracting financial data, which provides quantitative and qualitative information on Spanish companies. However, it was not possible to access the financial data for all sheltered employment centres. So, finally, the financial statements of 958 sheltered employment centres were collected from 2004 to 2016. This data represents 52% of all sheltered employment centres in Spain. This is the final sample of our research.

### Variables

The variables used were those that allow the analysis of the current situation of sheltered employment centres.

Our dependent variable is a viability measurement. We use the proxy of return on assets (ROA) because ROA is considered a ratio required for the financial viability of firms (Suarez 2000; Retolaza et al. 2014). In theory, like other firms on the market, sheltered employment centres should be profitable in order to survive but this is not their main goal but rather social implications. Furthermore, ROA has been used for other researchers to define the profitability of firms (José et al. 1996; Suarez 2000; García-Teruel and Martínez-Solano 2007; Isaac et al. 2009; Enqvist et al. 2014; Kohlscheen et al. 2018). The sheltered employment centres of our sample have been classified into two groups according to their ROA (operating result/total assets) values to carry out our analyses. Therefore, the ones that have a positive ROA value will be assigned to class 1. On the contrary, the sheltered employment centres with negative ROA values have been assigned to class 0.

According to most classical studies, profitability, cash flow, liquidity, leverage and efficiency ratios are the most used in failure prediction studies (Dimitras et al. 1996), which is the opposite concerning viability. However, as we want to explain profitability and not failure prediction, we just select the ones not correlated with ROA. First, a liquidity ratio is chosen. Another ratio not correlated with the dependent variable is the level of indebtedness. Additionally, the financial risk and growth rate in sales are also included. The financial risk ratio measures the ability of the company to cover its financial expenses through total sales (Serer et al. 2009; Tarrés 2012). This ratio is considered one of the most important to measure the economic and financial situation of the company. Sales growth is considered the main variable to check the company´s performance (Morbey and Reithner 1990). Several investigations have studied the relationship between company profitability and sales and have concluded that there is a positive relationship between the company's ROA and different types of sales (Gill and Mathur 2011; Kouser et al. 2012).

The list of the independent variables for regression is shown in Table 1.

**Table 1:** Independent variables

ID	Variables	Definition
AGE	Age	The number of years since its foundation
SIZE	Size	The number of employees
SECTOR	Sector of activity	Manufacturing companies 0; service companies 1
LOCAT	Location	Autonomous community (autonomous communities with the highest concentration of sheltered employment centres 1; otherwise 0)
LIQUID	Liquidity (quick ratio)	(Current assets-inventory)/current liability
INDEBT	Indebtedness	Total liabilities/total equity
F_RISK	Financial Risk	Financial expenses/sales
SALESGR	Sales Growth	Sales t –sales t-1/sales t-1
SALESEMP	Average sales per Employee	Sales/number of employees
SUBS	Subsidies (capital grants)	Amount of money received from public institutions
EF_CRISIS	Economic and Financial crisis (years)	During 2008–2014 = 1; 0 = otherwise

Source: own elaboration

Other variables that could condition the profitability of firms are sector, size and age. According to Claver et al. (2002), the profitability of a company is linked to industry. This variable has been classified according to the NACE (Statistical Classification of Economic Activities in the European Community) classification. After that, they have been divided into two parts: manufacturing activities and service activities. The division of this variable into two groups is mainly since most of the sheltered employment centres operate in the service sector (Jordán de Urríes and Verdugo 2010; Gelashvili et al. 2016). Additionally, many papers evidence the relationship between size and ROA although the results are not yet conclusive. Some papers justify a positive relationship (González Perez 1997; Pervan and Visic 2012), others a negative one (Antón et al. 1990) and, finally, the rest a neutral one (Galán and Vecino 1997). Location is also another important factor to take into account; this variable has been categorised and divided into autonomous communities with the highest concentration of sheltered employment centres and autonomous communities for a lower concentration of these companies. Based on the study elaborated by Gelashvili et al (2015b) higher number of sheltered employment centres were registered in autonomous communities such as Madrid, Catalonia, Andalusia and Castilla-León. In this way, we would like to know if location conditions the profitability of these companies because there is a different regulation about grants and subsidies for this type of company for each autonomous region. Finally, to answer research question 2, a dummy variable economic and financial crisis has been created. The economic and financial crisis in Spain started in 2008, when the overpricing of real estate assets played an important role in the process of weakening the banking system (Montalvo 2009; Alonso 2013; Bank of Spain 2017). Therefore, the sample has been divided into two parts, before and after the economic and financial crisis and the period of the crisis. That allowed us to see if the profitability of these companies has been affected by the economic and financial crisis or not.

### Methodology

We perform a detailed descriptive analysis of the sample to characterise the variables under study. Additionally, we use a correlation table in order to analyse the relationship between variables. We then generate a residual plot to verify the assumption of normality. To test our research questions, we build a multiple linear regression model that has the ROA of sheltered employment centres as a response variable and the various explanatory variables listed in Table 1. Taking into account that we have different types of variables, we use linear regression with random effects. Regression with random effects is a regression model (panel data) that combines cross-section and time-series data (Baltagi 2008; Hsiao 2014), as is the case of our model. To ensure that this was the right model, the Breusch-Pagan test has been carried out to determine whether we should perform the data analysis using the random effect or pooled estimation (Breusch and Pagan 1980). Following the research question (RQ1), we expect some variables to be significant, that is, lie below the level of significance of 5% (as p-value < 0.05). From the second research question (RQ2), we would expect the coefficient β of the year dummy variable to be significant as well (p-value < 0.05). This will mean that the economic and financial crisis will have an impact on the profitability of sheltered employment centres.

Additionally, to give another view of the problem raised in this work and complete the results obtained, a decision tree based on artificial intelligence (AI) methodology has been developed. Methods based on AI are widely used to analyse financial problems (Serrano and Martin del Brio 1993; Sanchis et al. 2007; Diaz et al. 2009). Indeed, AI methods are a complement and, in some cases, a substitute for statistical methods. In any case, they can give another point of view on the problems we are investigating. Consequently, we are going to examine the economic and financial variables that characterise failure, and therefore, the survival of sheltered employment centres using the C4.5 decision tree. Several algorithms develop decision trees, and what differentiates one decision tree from another is the algorithm that generates it. The algorithm developed by Quinlan and implemented in C4.5 (Quinlan 1993) is probably the most popular of all decision tree algorithms. In it, the criterion used to make the partitions is based on a series of Information Theory concepts and has experienced a series of notable improvements over time. For a more detailed description, see Quinlan (1993) and Díaz et al. (2009).^4^

## Results and discussion

### Linear regression result

The results of the descriptive analysis show that sheltered employment centres, on average, are companies with a positive rate of profitability (see Table 2). It is highlighted that the average ROA is equal to 1.64%. This means that the assets of sheltered employment centres generate enough profit, although it is not very high. The average age of sheltered employment centres is more than 10 thus, the majority of them are companies with experience in the market. The size of sheltered employment centres has been measured by the number of employees. The average number of employees for these companies is 56. This means that these kinds of firms are very labour intensive.

**Table 2:** Descriptive statistics

Variable	Obs	Mean	Std. Dev	Min	Max
ROA	9083	1.64	20.21	− 253.46	617.41
AGE	9083	10.24	6.83	1.00	52.00
SIZE	9083	56.58	142.13	1.00	2338.00
LIQUID	9083	2.75	47.68	0.00	4164.31
INDEBT	9083	3.43	72.19	− 393.01	6304.48
F_RISK	9083	− 0.27	3.73	− 53.15	172.57
SALESGR	9083	0.42	8.77	− 1.00	659.91
SALESEMP	9083	51,266.74	316,197.80	0.00	20,800,000.00
SUBS	9083	100,483.70	590,708.10	0.00	10,500,000.00

Source: own elaboration

The result of the descriptive analysis has shown that the average of public subsidies is 100,483 euros. That means that the companies that receive public subsidies usually get a fairly high amount of funds. The maximum amount in terms of subsidies received per company is 10 million, while almost half of these companies have not received these subsidies. By analysing the liquidity ratio it can be observed that its average is 2.75, indicating that sheltered employment centres can pay off their short-term obligations. The result of the indebtedness ratio shows that sheltered employment centres are heavily indebted, 3.43, and 100 euros net worth, on average, meaning they have 343 euros of debts. The negative result of the financial risk ratio indicates that the level of financial risk of sheltered employment centres is high, even though its standard deviation is also high. The positive results of the growth in sales of variables and sales per employee indicate that these companies, on average, have a positive return on sales.

The descriptive analysis that gives the first image of the economic and financial situation of the companies was followed by the correlation matrix. The link between ROA and the independent variables is shown in Table 3. There is an interesting correlation between ROA and the size of the company. The profitability of sheltered employment centres is also determined by their business activity. Correlation between the dependent variable and the age of sheltered employment centres, indebtedness, financial risk and economic and financial crisis is also shown.

**Table 3:** Correlation matrix

	ROA	AGE	SIZE	SECTOR	LOCAT	LIQUID	INDEBT	F_RISK	SALESGR	SALESEMP	SUBS	EF_CRISIS
ROA	1											
AGE	− 0.0269*	1										
	0.010											
SIZE	0.0583*	0.2863*	1									
	0.000	0.000										
SECTOR	0.0357*	− 0.0638*	0.0751*	1								
	0.001	0.000	0.000									
LOCAT	0.002	− 0.002	0.0410*	0.1183*	1							
	0.827	0.836	0.000	0.000								
LIQUID	− 0.009	− 0.003	− 0.007	0.014	0.017	1						
	0.398	0.760	0.489	0.198	0.106							
INDEBT	− 0.0275*	− 0.014	− 0.009	0.009	0.010	0.000	1					
	0.009	0.189	0.374	0.384	0.361	0.972						
F_RISK	− 0.0658*	− 0.0213*	− 0.003	− 0.006	− 0.0289*	0.004	− 0.001	1				
	0.000	0.043	0.810	0.551	0.006	0.697	0.929					
SALESGR	0.010	− 0.021	− 0.013	0.015	0.018	− 0.001	0.001	− 0.0018	1			
	0.342	0.050	0.223	0.161	0.090	0.946	0.927	0.8607				
SALESEMP	0.014	0.0479*	− 0.0388*	0.013	0.001	− 0.003	0.003	− 0.0147	0.02	1		
	0.197	0.000	0.000	0.227	0.910	0.803	0.799	0.1604	0.0564			
SUBS	0.003	0.2398*	0.3854*	0.006	− 0.0284*	0.001	− 0.006	0.0230*	− 0.005	0.0306*	1	
	0.796	0.000	0.000	0.576	0.007	0.940	0.599	0.0285	0.6311	0.0035		
EF_CRISIS	− 0.2757*	0.1888*	0.013	0.0363*	0.007	0.0300*	0.0246*	− 0.0379*	0.0254*	0.0844*	0.0885*	1
	0.000	0.000	0.213	0.001	0.500	0.004	0.019	0.0003	0.0153	0.000	0.000	

Source: Own elaboration

Another interesting point is that there is no correlation between the profitability of these companies and public subsidies as capital grants. This means that the idea that the viability of sheltered employment centres is due to their public subsidies is not true based on the correlation table result. However, the results of the correlation are not conclusive.

The next step in our statistical analysis was the elaboration of linear regression for panel data. A total of 953 groups were analysed with 9,083 observations. The result has shown that the explanatory variables explain an overall 87% of the proposed model and 93% within groups. Furthermore, the p-value was 0.00. This allows us to affirm that the independent variables chosen have explanatory power for the model. In Table 4 we can see that the linear regression confirms the result of the correlations, where there is a positive relationship between the dependent variable and the size of the company. Therefore, our results coincide with the statement of González Perez (1997) and Pervan and Visic (2012). The dummy variable that classifies sheltered employment centres by sector of activity is not significant (p-value = 0.369 > 0.05), although, the correlation table showed that there was a correlation between ROA and the sector of activity of these social companies. That means that the profitability of sheltered employment centres does not depend on the sector in which they operate. The age of sheltered employment centres is one of the variables that explain the proposed model. But, the coefficient of this variable is negative. This means that new companies have more profitability than the companies with experience at the market. Also, the size of these companies and financial risk are the variables that condition their profitability. The results achieved by González Perez (1997), Claver et al. (2002) and Pervan and Visic (2012) are in line with the results of this study.

**Table 4:** Results of the linear regression

ROA	Coef	Robust Std. Err	t	P > t	[95% Conf. Interval]	
AGE	− .0093609	.0008883	− 10.54	**0.000**	− .0111019	− .0076199
SIZE	.0002296	.000048	4.78	**0.000**	.0001354	.0003237
SECTOR	.0159065	.0177012	0.90	0.369	− .0187872	.0506003
LOCAT	− .0049561	.015046	− 0.33	0.742	− .0344456	.0245335
LIQUID	− .0000233	.0000828	− 0.28	0.778	− .0001857	.000139
INDEBT	− .0000744	.0000541	− 1.37	0.169	− .0001805	.0000317
F_RISK	− .0071229	.0010577	− 6.73	**0.000**	− .0091959	− .0050498
SALESGR	.0009191	.0004478	2.05	**0.040**	.0000414	.0017968
SALESEMP	2.13e− 08	1.28e−08	1.67	0.094	− 3.67e−09	4.64e−08
SUBS	8.76e−09	1.00e−08	0.87	0.382	− 1.09e−08	2.84e−08
EF_CRISIS	− .1788339	.0079041	− 22.63	**0.000**	− .1943256	− .1633421
_cons	.9434982	.0190962	49.41	0.000	.9060703	.9809261

Source: Own elaboration

Other variable that explain the ROA of sheltered employment centres is sales growth. This result confirms the findings of Gill and Mathur (2011) and Kouser et al. (2012), although it should be noted that these studies have analysed the sample of companies operating in different countries such as Canada, where company policy and operation are different. Therefore, this result is an important finding for Spanish social firms and especially for sheltered employment centres, since no prior studies were linking the profitability of sheltered employment centres to sales, especially when it is questioned if the profitability of these companies is related to the subsidies received. The correlation between the explanatory variable and these variable has also been confirmed in Table 3.

To answer research question 2, the independent variable “economic and financial crisis” has been introduced into the model. To measure the effect of the economic crisis on the profitability of these companies, the linear regression has been performed, considering the period of crisis in the years 2008–2014. Therefore, dummy variable (EF_CRISIS) is created where the non-crisis period were 2004–2007 and 2015–2016 as zero and the economic and financial crisis period from 2008–2014 as one. We have made this division based on the GDP growth in Spain. According to data provided by the INE (2020),^5^ in the years of financial crisis, Spain's GDP decreased considerably, although in 2015 it has begun to recover. Bank of Spain (2017) has pointed out that the economic and financial crisis in Spain is understood between the years 2008–2014.

The results of the linear regression for panel data indicate that the ROA of sheltered employment centres is explained by the variable economic and financial crisis. However, it should be noted that the coefficient that explains this relationship is negative. The negative coefficient suggests that, as the independent variable “ROA” increases, the dependent variable “economic and financial crisis” tends to decrease. During the economic and financial crisis, the profitability of these companies was lower than in the other years. Therefore, we can say that sheltered employment centres like the rest of the companies in Spain have been affected by the crisis.

The following section studies the proposed model by using the C4.5 decision tree.

### C4.5 decision tree

The model to support our results of the main analysis has been compiled through the C4.5 decision tree. As the sample in this article is from panel data, two different years have been analysed, namely 2008 and 2016. 2016 has been used as the last year of the sample and 2008 as the year when the economic and financial crisis started in Spain.

For 2008, 643 sheltered employment centres were used. The result has shown that almost 91% of the sample was correctly classified. Data for the dependent variable (ROA) was classified into two groups: group 0 classifies sheltered employment centres with a negative return on assets and group 1 with positive results.

**Fig. 1:**
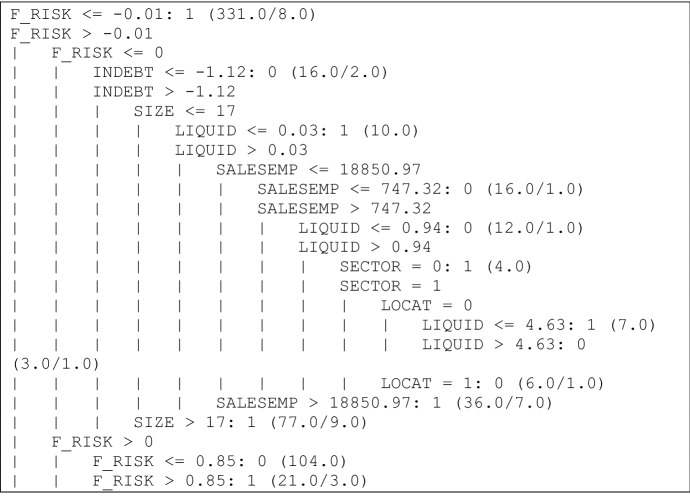
Decision tree obtained by algorithm C4.5, 2008 (I). *Source* Own elaboration

The results of the model can be seen in Fig. 1. The strongest branches for each class (0, 1) have been highlighted in grey, i.e. those that verify a greater number of sheltered employment centres. The independent variable such as economic and financial crisis was not examined, since by 2008 all companies were adopting zero and by 2016 one.

**Fig. 2:**
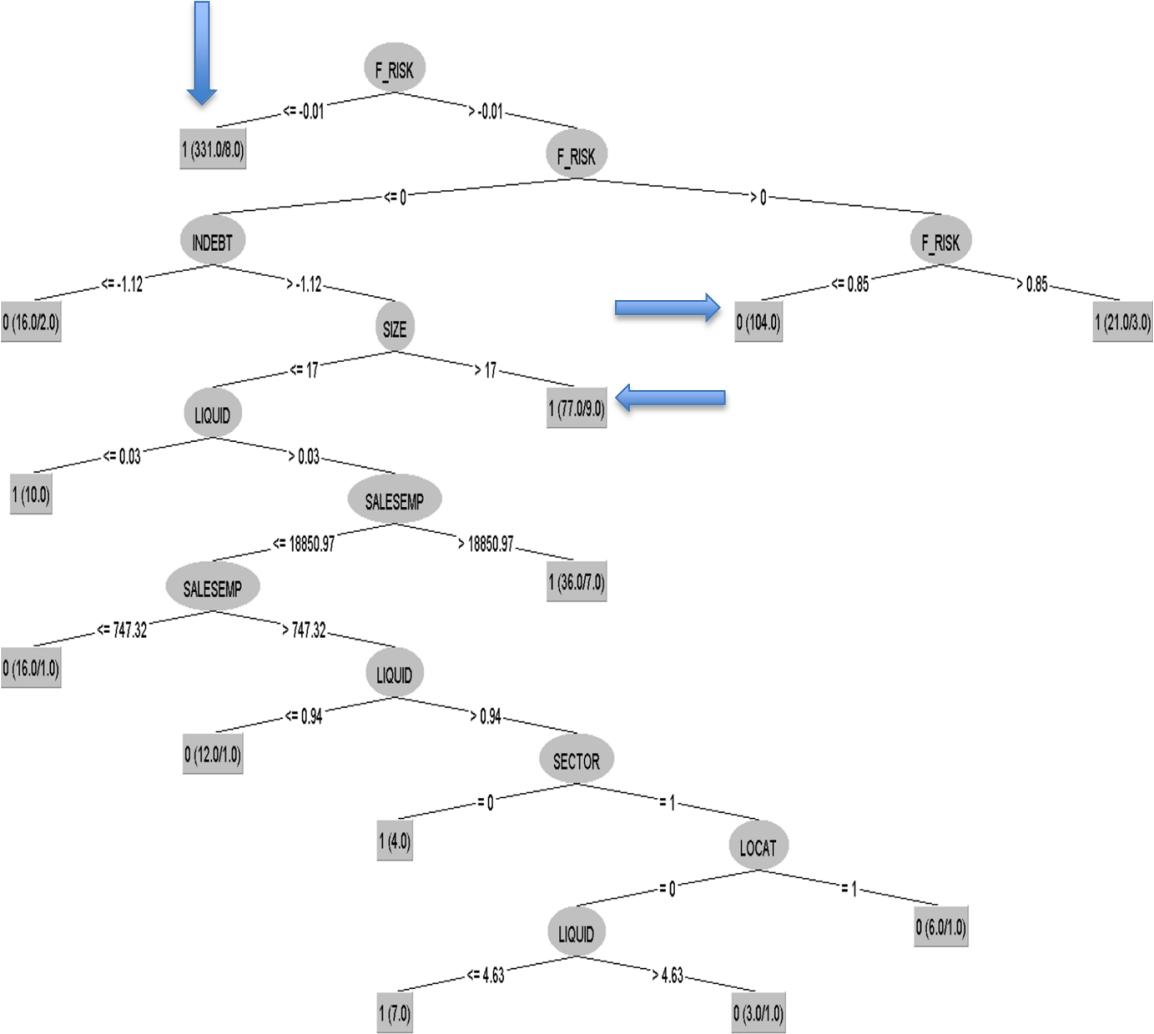
Decision tree obtained by algorithm C4.5, 2008 (II). *Source* Own elaboration. The strongest rules are pointed out with an arrow

The branches with more sheltered employment centres are those that should be interpreted since they would reflect certain patterns as they are supported by a large number of cases. As it can be observed in the graph, the most important branch that classified profitable sheltered employment centres indicates that if the financial risk ratio of these companies is less than or equal to − 0.01, the sheltered employment centres are profitable companies (this branch is supported by 331 firms, with 8 errors). Taking into account profitable firms, another strong branch shows that if the financial risk ratio is between − 0.01 and 0, the indebtedness ratio is greater than − 1.12 and the number of employees is more than 17, the sheltered employment centres are profitable (this branch is verified by 77 firms, with 9 errors). Finally, another important branch matches the following rule: if the values of financial risk ratio are between − 0.01 and 0.85, the sheltered employment centres are non-profitable firms (this rule is supported by 104 companies). Therefore, according to this method, the financial risk ratio is a key variable (Fig. 2).

**Fig. 3:**
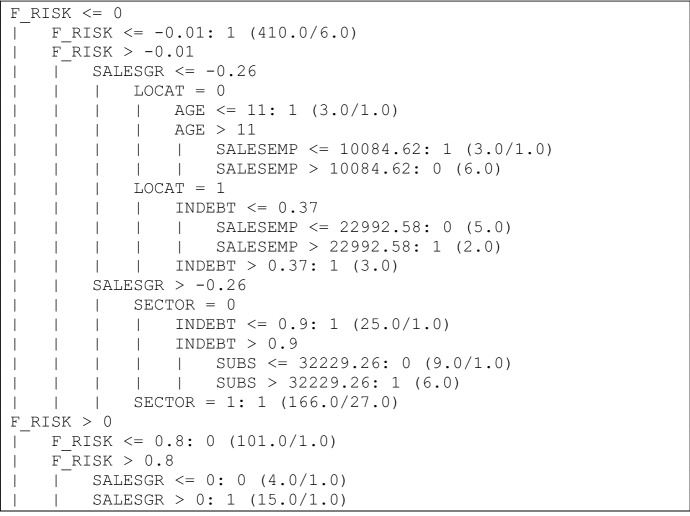
Decision tree obtained by algorithm C4.5, 2016 (I). *Source* Own elaboration

**Fig. 4:**
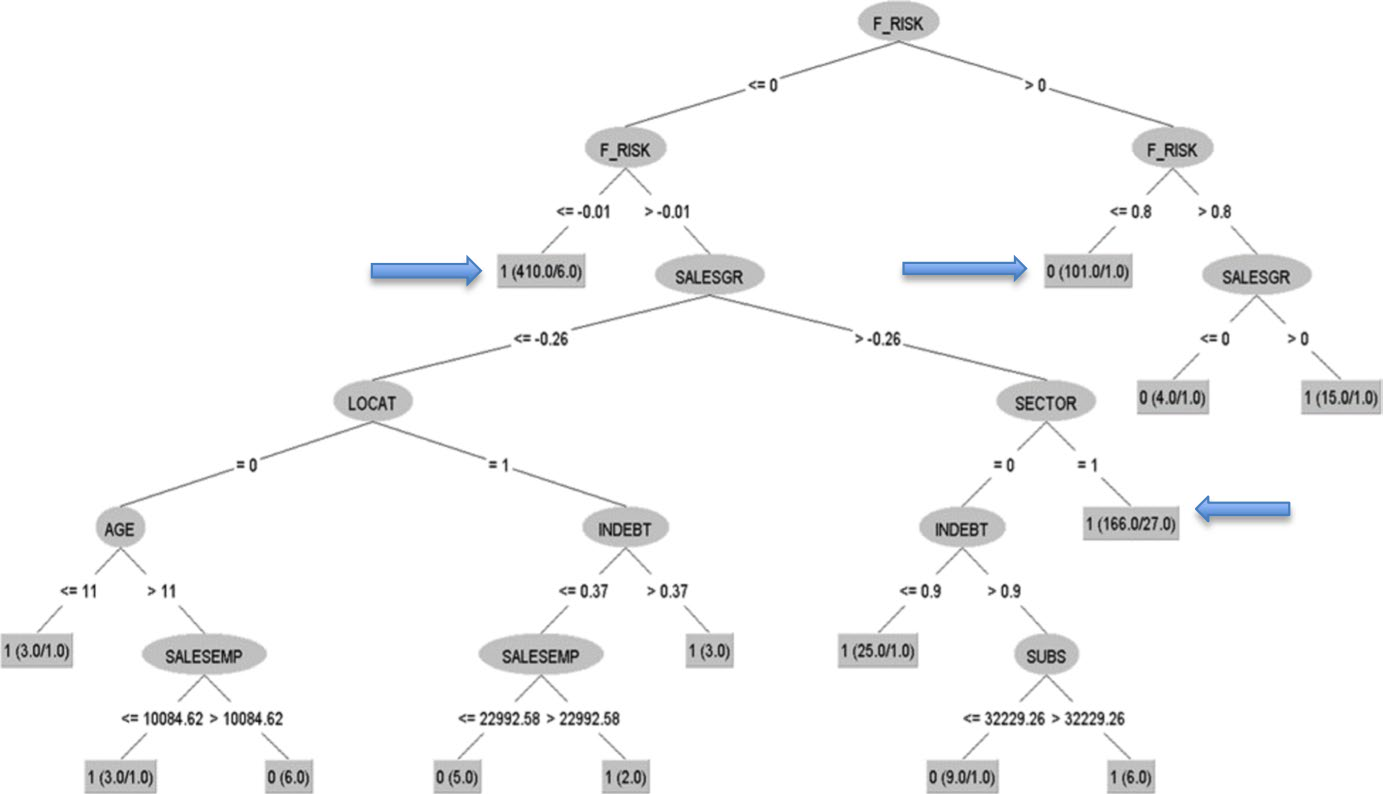
Decision tree obtained by algorithm C4.5, 2016 (I). *Source* Own elaboration. The strongest rules are pointed out with an arrow

The next step is to see which rules qualify these companies to be profitable or not in 2016. For that year it was possible to analyse 758 sheltered employment centres. The good results in terms of cross-validation (91% classified correctly) justify the analysis of the patterns shown in the tree. Therefore, Figs. 3 and 4 are presented.

As the results show, there are two strong rules for class 1. The first rule shows that if the financial risk ratio of these companies is less than or equal to − 0.01, then the sheltered employment centres are profitable firms (this rule is supported by 410 firms, with six errors). The second rule shows that if the financial risk ratio of these companies is greater than − 0.01, the sales growth ratio is greater than − 0.26 and is a service company, then the sheltered employment centres are profitable firms (this rule is supported by 166 firms, with 27 errors). For non-profitable firms, the tree shows a strong rule with the following pattern: if the financial risk ratio is positive but lower than or equal to 0.8, then the sheltered employment centres belong to the non-profitable category (this pattern is satisfied by 101 firms with one error). Similarly to the previous decision tree, the key variable is the financial risk ratio.

## Conclusions

This study aimed to investigate the financial viability of all the sheltered employment centres operating in Spain through different accounting ratios. The results of the descriptive analyses show that sheltered employment centres are companies with a low rate of profitability, but their financial risk is low. The explanation could be that they are social firms looking for the labour integration of disabled people, without maximising their net income or incurring high risk. However, minimum profitability is needed to survive in the market.

Based on the regression analysis and decision trees, the key variable for the profitability of these companies is financial risk. This variable analyses the relationship between the company’s financial expenses and sales and shows whether or not it is financially stable. Therefore, we can say that sheltered employment centres that can meet their financial expenses with their main income can have optimal profitability, as once the profitability of these companies increases their financial risk decreases.

The variables like the sales growth for these companies is significant in the regression analysis. This shows that these companies are working hard to carry out their main activity, achieving profitability and viability through it. Although this result has not been confirmed in the decision tree results, we must take into account the time horizon examined in decision trees, which is one year, and in regression, we have worked with panel data for 12 years. The result of correlations and linear regression have shown that the variable economic and financial crisis is a key factor in explaining the profitability of sheltered employment centres. Outcomes have shown that the profitability of these companies was lower during the economic and financial crisis. Even so, we could not verify this result in the decision tree analysis, since this variable was the dummy variable, which we have not managed to analyse through AI. Another variable that is important for the profitability of these companies is the size.

Because of the above, we can say that the managers of these companies have to take special care of those factors such as financial risk, size and sales growth (these last two are not confirmed by the results of the decision trees, but they are confirmed by the regression and correlation analysis), if they want to ensure the future viability of these companies. In addition to this, the governments of each region must invest more in equal opportunities for people with disabilities. In this sense, the sheltered employment centres are companies whose main challenge is to ensure paid work for people with disabilities and, at the same time, to stay in the market.

Finally, we consider that the creation of employment for people with disabilities is highly significant for society in general as well as for the economy of the country. In addition, social and labour integration helps people with disabilities have less social, mental, medical and financial problems. Therefore, the role of the sheltered employment centres is essential, especially in the current or future time of crisis that is destroying and will destroy millions of workplaces. Although the results show that the profitability of these companies has been affected in the time of the past economic and financial crisis, they have managed to survive and maintained employment for people with disabilities. This shows the importance of these companies, which can help change the world even during the economic and financial crisis.

This study is not free of limitations. One of the limitations is the lack of up-to-date data, especially economic and financial data, and the complete list of sheltered employment centres. Up until now, there is no complete list of how many sheltered employment centres there are in Spain. Several autonomous communities publish their provisional list, but many do not, and this makes it difficult to find the data. The results obtained in this study can be a good basis in the future, when financial data will be available for all sheltered employment centres, to show if there are significant changes in their economic-financial structure. Consequently, the lack of current economic and financial data for these companies is one of the important limitations. Another limitation of the study is not being able to use more independent variables. Therefore, for future research lines, we would like to investigate all sheltered employment centres, updated by the last year available, taking into account a wide range of already-existing ratios and new ones like a political party that governs an autonomous community. Apart from this, future research would entail analysing all social enterprises (sheltered employment centres, social cooperatives and insertion companies) in Spain to see what the main similarities and differences are between them. The analysis of data through other AI algorithms and statistical techniques for panel data is also proposed. Therefore, our main future research line would be to conduct a more in-depth study of these important topics in an attempt to present the updated financial data for social firms and especially for sheltered employment centres.

## Footnotes

1 Ministry of Industry, Commerce and Tourism. SME reports 2006, 2015. http://www.ipyme.org/es-ES/AreaEstadisticas/Paginas/InformesPYME.aspx.

2 https://www.lamoncloa.gob.es/serviciosdeprensa/notasprensa/trabajo14/Paginas/2020/050520-paro.aspx.

3 http://www.bvdinfo.com/en-uss/our-products/company-information/national-products/sabi.

4 The J48 algorithm is the freely accessible Java implementation of the C4.5 algorithm contained in the WEKA software. In fact, WEKA is the data mining package developed by the University of Waikato (Witten and Frank 2005) with which we have performed our analysis to complete the main result.

5 Instituto Nacional de Estadistica (INE)—[Spanish Statistics Institute]. Available at: https://www.ine.es/dyngs/INEbase/es/operacion.htm?c=Estadistica_C&cid=1254736167628&menu=resultados&idp=1254735576581#!tabs-1254736158133.
